# The Role of Inflammation in Venous Thromboembolism

**DOI:** 10.3389/fped.2018.00142

**Published:** 2018-05-23

**Authors:** Brian R. Branchford, Shannon L. Carpenter

**Affiliations:** ^1^University of Colorado Hemophilia and Thrombosis Center, Pediatric Hematology, University of Colorado School of Medicine and Children's Hospital Colorado, Aurora, CO, United States; ^2^Kansas City Regional Hemophilia Treatment Center, Pediatric Hematology, UMKC School of Medicine and Children's Mercy Hospital, Kansas, CO, United States

**Keywords:** pediatrics, venous thromboembolism, thrombosis, inflammation, cytokines, platelets, risk factors

## Abstract

Venous thromboembolism (VTE), comprising deep vein thrombosis (DVT), and pulmonary embolism (PE), is becoming increasingly recognized as a cause of morbidity and mortality in pediatrics, particularly among hospitalized children. Furthermore, evidence is accumulating that suggests the inflammatory response may be a cause, as well as consequence, of VTE, but current anticoagulation treatment regimens are not designed to inhibit inflammation. In fact, many established clinical VTE risk factors such as surgery, obesity, cystic fibrosis, sepsis, systemic infection, cancer, inflammatory bowel disease, and lupus likely modulate thrombosis through inflammatory mediators. Unlike other traumatic mechanisms of thrombosis involving vascular transection and subsequent exposure of subendothelial collagen and other procoagulant extracellular matrix materials, inflammation of the vessel wall may initiate thrombosis on an intact vein. Activation of endothelial cells, platelets, and leukocytes with subsequent formation of microparticles can trigger the coagulation system through the induction of tissue factor (TF). Identification of biomarkers to evaluate VTE risk could be of great use to the clinician caring for a patient with inflammatory disease to guide decisions regarding the risk:benefit ratio of various types of potential thromboprophylaxis strategies, or suggest a role for anti-inflammatory therapy. Unfortunately, no such validated inflammatory scoring system yet exists, though research in this area is ongoing. Elevation of C-reactive protein, IL-6, IL-8, and TNF-alpha during a response to systemic inflammation have been associated with increased VTE risk. Consequent platelet activation enhances the prothrombotic state, leading to VTE development, particularly in patients with other risk factors, most notably central venous catheters.

Evidence is accumulating that the factors influencing VTE formation are not restricted to the coagulation system alone, but rather that the immune system is also closely involved with formation and resolution of thrombosis. It is important to consider the contributions of inflammation to VTE development in general, but also as it pertains to certain specific disease categories more commonly associated with development of hospital-acquired VTE (HA-VTE) in some patients. Surgery, (1–3) obesity, (4) cystic fibrosis, (5–7) sepsis, (8–10) systemic infection, (11, 12) cancer, (13–15) inflammatory bowel disease, (16–18), and lupus (19–22) are clinical VTE risk factors that may modulate thrombosis through inflammatory mediators. Understanding the role of inflammation in these particular clinical situations may not only help determine the optimal management but may also aid in the development of future preventative strategies, since current anticoagulation treatment regimens are not designed to inhibit inflammation (23).

Recently, inflammation has been accepted as a common pathway through which various risk factors trigger VTE formation. A feasible mechanism is that inflammation of the vessel wall initiates thrombus formation in an intact vein and that inflammation and coagulation systems are coupled by a common activation pathway. The first event in thrombus formation is probably activation of endothelial cells, platelets, and leukocytes, with initiation of inflammation and formation of microparticles that trigger the coagulation system through the induction of TF. Therefore, the key event in the initiation of VTE formation is most likely vein wall inflammation, but the contribution of specific immune modulators has not yet been elucidated. Recently, it was demonstrated that probable association between VTE and several other markers of inflammation such as C-reactive protein (CRP), IL-6, IL-8, and tumor necrosis factor-alpha exists (24–28). These pro-inflammatory cytokines play an important role in VTE by promoting a pro-coagulant state primarily by inducing the expression of tissue factor. Several immune system components (cytokines, chemokines, and various leukocyte subtypes) are involved in the underlying inflammatory process of VTE, as is very well-described in a recent review from Saghazadeh et al. (23). Additionally, it has been recently described that inflammatory mediators such as polyphosphates, bradykinin, and others may directly activate the contact system (the polyphosphate—Factor XII association is particularly notable) and initiate the extrinsic coagulation pathway (29–31).

The identification and elucidation of inflammatory markers relevant to VTE could provide targets for future therapy. That inflammation is the basic etiopathogenic process of VTE is also supported by the relation of some risk factors to both arterial and venous thrombosis: age, increased BMI, atherosclerotic disease, hypercholesterolemia, hypertension, antiphospholipid antibodies, and hyperhomocysteinemia (28 ).

## Interaction between coagulation and inflammation

For over a century, thrombosis formation has been attributed to three main groups of factors including alterations in blood flow, endothelial injury, and hypercoagulable state, collectively known as Virchow's Triad (32). A prime example is the frequent association of central venous catheter (CVC) with VTE. Blood flow is altered by the obstacle in the vein, creating turbulent flow in some areas, while promoting stasis in others, depending on local vascular architecture. Additionally, the endothelium is damaged at the catheter insertion site, and the body actively attempts to repair that site with primary and secondary hemostatic efforts. Finally, there is a high likelihood that the underlying disease process that necessitated the need for the CVC in the first place (volume resuscitation in trauma or distributive shock, chemotherapy administration for active cancer, long-term antibiotics for severe infection, etc.) increases the risk for thrombosis. There is also likely a reciprocal relationship between CVC infections leading to increasing risk for line thrombosis and infected clots within a line likely to adversely affect its function. Over the last decade, a growing body of evidence suggests a role for consideration of inflammation as a major contributor to the pathophysiology of VTE, (33) likely by enhancing the hypercoagulable state and increasing endothelial damage. Activation of endothelial cells, platelets, and leukocytes, with subsequent initiation of inflammation and microparticle formation, triggers the coagulation system through induction of TF, primarily that borne by microparticles, which may contribute to the hypercoagulable state in the Triad (28, 34, 35). Both formation and resolution of thrombosis have been associated with a series of inflammatory cascades (36, 37). Moderate to severe degrees of inflammation (inflammatory infiltrates throughout the thrombi, mostly composed of lymphocytes, with some mixing of other components including plasma cells, neutrophils, and eosinophils) (38) were found in approximately 15% of thrombus specimens from pulmonary thromboendarterectomy (23) and immunity/inflammatory genes constitute nearly 10% of those genes whose expressions are substantially altered under the influence of VTE (38, 39). A reciprocal relationship exists in which patients with VTE have an increased risk of myocardial infarction and stroke, and vice versa, while proven cardiovascular risk factors such as obesity, tobacco use, diabetes, stress, and diet increase the risk of both atherothrombotic events and VTE, likely due to the common pathophysiological mechanism of promoting cardiovascular inflammation (40).

Evidence of specific pathophysiologic links between the coagulation system and innate immunity continues to mount. Polyphosphate (polyP) is present in human platelet dense granules and is released upon platelet activation, assisting with coagulation by increasing activation of factor V, decreasing tissue factor pathway inhibitor (TFPI) activity, and delaying clot lysis by activating thrombin-activatable fibrinolysis inhibitor (TAFI) (41–44). PolyP is also a potent pro-inflammatory signal when released from mast cells during a hypersensitivity reaction, for example (42).

Additionally, histones have been shown to be increased in sepsis and other inflammatory conditions along with nucleosomes (DNA + histones), and are toxic to the endothelium. Activated protein C inactivates histones, protecting the endothelium in the process (45). Extracellular DNA fibers extruded from neutrophils (Neutrophil Extracellular Traps, NETs) are produced in response to infection to allow neutrophils to trap and destroy invading microorganisms. Fibrin formation and deposition has been shown to be stimulated by NETs, and fibrin formation is also important in trapping organisms and controlling infection (46). NETs also cause platelet adhesion and have been shown to be linked to deep vein thrombosis in experimental models (47). Moreover, platelets have been shown to stimulate NET production (46). There is also evidence that some bacteria interact with platelets, either directly or through antibody-mediated mechanisms, leading to platelet activation or contributing to thrombus development. Other interactions have been identified which are beyond the scope of this brief review and have been described excellently elsewhere (45, 48–50).

Inflammation can be both a cause and a consequence of VTE, but current anti-coagulation treatment regimens are not specifically designed to inhibit inflammation (though it is known that heparins have some anti-inflammatory effects). Selective pharmacologic targeting of immune/inflammatory mediators in VTE may result in more effective therapeutic or prophylactic strategies (23). It is important to note, however, that the role of anti-inflammatory therapies in VTE prevention efforts is still not well-established. One study demonstrated a 2-fold or more increased risk of VTE with the use of non-selective non-steroidal anti-inflammatory drugs (NSAIDs) or cyclooxygenase-2-selective (COX-2) inhibitors (51). These results were reflected in a recent systematic review and meta-analysis in which the pooled risk ratio among NSAID users was 1.8-fold for VTE (52). Adequate VTE mechanism data in this setting are not yet available, however, to determine whether the anti-inflammatory medication itself drives the VTE formation or whether inflammation drives the usage in patients who are being treated with anti-inflammatory drugs to manage their underlying medical condition. The same question may be considered in users of steroids, though it has been shown more specifically that glucocorticoids increase levels of clotting factors and fibrinogen, which may explain the elevated VTE risk (especially pulmonary embolism) demonstrated in a recent Danish study of glucocorticoid users that followed an associated temporal pattern, persisted after adjustment for underlying disease severity, and existed even in non-inflammatory conditions (53). A similar effect with 1.5-fold increased VTE risk was seen in subjects treated with glucocorticoids for at least 30 days prior to surgery (54).

Particular disease subgroup-specific relationships have been studied, as seen below and in Table 1.

**Table 1:** Inflammatory considerations in specific clinical situations.

**Clinical subgroup**	**Inflammatory considerations**
Surgery	The healing process is, at baseline, inflammatory since this is the mechanism by which tissues are re-approximated and cellular debris is cleared. Surgery may also be in response to an inflammatory stimulus (e.g., cancer, abscess, trauma). SCD usage may be helpful in surgeries extending over a certain time duration because of their mechanism of replacing the venous return assistance usually provided by the body's lower extremity muscle contractions while walking.
Inflammatory bowel disease	Various reports of odds ratio between 1.5 and 3.5 for increased VTE risk in patients with IBD. Primary risk factors in this subgroup include disease severity, colonic localization, and recent surgery. Risk decreases with treatment of underlying condition.
Obesity	Obesity confers an odds ratio between 1.7 and 2.2 for VTE, likely acting through higher CRP levels found in subjects with higher BMI, in addition to potential for decreased physical activity.
Cystic fibrosis	Multifactorial increased VTE risk: frequent hospitalizations, systemic or severe local infections (especially with specifically thrombogenic strains such as *B. cepacia*), elevated acute phase reactants, CVC presence, activated platelets. Risk decreases with appropriate management of underlying condition.
Sepsis/systemic infection	Specific risk from acute phase reactant release (tissue factor, VWF, procoagulant microparticles), inhibition of fibrinolysis, NET formation, etc. Risk is increased in both systemic and severe local infections.
Systemic lupus erythematosus	Particularly challenging due to existence of both chronic and acute inflammatory-driven risk states and auto-immune component (APLA, innate immune dysregulation, etc.). Risk decreases with treatment of underlying condition.

## Surgery and trauma

Surgery and trauma are linked in terms of HA-VTE risk due to the tissue injury they have in common. The role of inflammation in driving HA-VTE risk in these clinical situations is primarily related to the process through which transected vessels and tissue undergo physiologic repair. The innate immune system is activated following tissue injury, platelets degranulate locally after recruitment from the circulation, and tissue macrophages and mast cells are activated with a combined effect of leukocyte chemoattraction (55). Neutrophils infiltrate the wound, followed by additional circulating monocytes that differentiate into mature tissue macrophages, (56) and later by additional infiltration of mast cells from adjacent tissue (57), and finally by T-lymphocytes (58). The collective result of these cellular activation processes is increased release of IL-1, IL-6, IL-12, and TNFα (59, 60). IL-6 has been demonstrated to play a critical role in inflammation-related thrombosis (61) increased concentrations of IL-6, along with TNFα and IL-8 are potent risk predictors for VTE, even after adjustment for covariates, including CRP (62). Additional neutrophil presence (following chemoattractant release from platelets and mast cells) and subsequent NET formation have been linked to venous thrombosis (47, 63). The interaction between endothelial E-selectin, leukocyte L-selectin, and platelet P-selectin also plays an important role in platelet-leukocyte aggregation and adherence to vessel walls at sites of surgical or traumatic injury. P-selectin inhibition has been shown to decrease venous thrombosis in murine (64) thrombosis models and enhance thrombus resolution in rat models (65). Similarly, E-selectin inhibition with a small molecule inhibitor has been shown to decrease inflammation (vein wall monocyte extravasation) and acute venous thrombosis in a surgical model of murine thrombosis induction (66) and is now being studied in early clinical trials in humans.

## Inflammatory bowel disease

Among VTE-related hospitalizations, the presence of IBD was associated with a 2.5-fold increased risk of mortality in one population-based study (18). No studies have specifically evaluated the potential benefit of VTE prophylaxis in hospitalized or ambulatory IBD patients, but studies to date do not support an increased bleeding risk with moderate doses of anticoagulant medications in IBD patients with active disease (18). Recent large studies have quantified this risk showing that IBD patients run a 1.5 to 3.6 higher risk of developing VTE than healthy controls (16, 17). IBD has been demonstrated to represent an independent risk factor for the recurrence of VTE (17).

IBD has been previously associated with increased levels of TNFα (67) with upregulation of TNFα mRNA in colonic tissue in patients with Crohn's Disease (68) and Ulcerative Colitis (69) and many current therapeutic regimens involve a focus on blocking signaling through this molecule. Giannotta et al. (16) have recently reviewed the specific effects of TNFα on activation of intrinsic coagulation pathway by inducing tissue factor expression on leukocyte surfaces, down-regulation of natural anticoagulants (protein C and heparin-antithrombin pathways) in addition to thrombomodulin and the endothelial protein C receptor, increasing platelet production and enhancing thrombin formation in conjunction with IL-6, and triggering (in conjunction with IBD-associated elevation in homocysteine) expression of vascular cell adhesion protein-1 and monocyte chemoattractant protein 1 on endothelial surfaces leading to enhanced capacity to recruit T cells and monocytes.

## Obesity

Obesity is a known risk factor for arterial and venous thrombosis. Studies have shown that obesity conveys an odds ratio of 1.7–2.2 for VTE, similar to that of other known VTE risk factors (70). A recent cohort of 268 adults with incident VTE events over 4.6 years from the Reasons for Geographic And Racial Differences (REGARDS) cohort demonstrated that higher CRP levels and lower serum albumin levels were associated with increased VTE risk and statistically mediated part of the association of body mass index (BMI) with VTE, suggesting that inflammation may be a potential mechanism underlying the relationship between obesity and VTE risk. White blood cell (WBC) count and platelet count were not determined to have any relationship. Adipocytes secrete inflammatory cytokines leading to chronic, low-grade inflammation resulting from recruitment of macrophages to adipose tissues that progressively accumulate as fat mass increases, and drive a progression of anti-inflammatory M2 macrophages to proinflammatory M1 macrophages, leading to increased secretion of proinflammatory cytokines including TNFα, IL-6, and IL-1β, as well as impaired fibrinolysis due to marked increase in plasminogen activator inhibitor-1 expression (70). These proinflammatory cytokines, as well as adipokines such as leptin, stimulate vascular endothelium, platelets, and other circulating vascular cells, leading to upregulation of procoagulant factors including tissue factor and cellular adhesion molecules, downregulation of anticoagulant regulatory proteins, increased thrombin generation, and enhanced platelet activation (71). Finally, aberrant microRNA expression patterns likely contribute to thrombosis in obesity through decreased endothelial nitric oxide bioavailability, unregulated expression of endothelial adhesion molecules, and enhanced platelet activation and degranulation as reviewed by Blokhin and Lentz (72).

## Cystic fibrosis (CF)

CF is a multi-system inflammatory disease, complicated by excessive production of thick mucus secretions that lead to pulmonary infectious exacerbations resulting in hospitalization for IV antibiotics, frequently through a CVC. These patients have increased risk of thrombosis due to CVCs, as well as acquired thrombophilia secondary to inflammation [including both the elevation of pro-coagulant acute phase reactant proteins such as fibrinogen, factor VIII, and/or von Willebrand factor (VWF), as well as the suppression of protein S by inflammation-related C4B binding protein], or natural anticoagulant protein deficiencies due to vitamin K deficiency and/or liver dysfunction (6). Also, the incidence of antiphospholipid antibodies that may be associated with increased thrombotic risk ranges from about 5–10% in patients with CF, which is elevated compared to that reported in healthy children (1–3%) (73, 74). While platelets are generally thought to play a more significant role in the pathogenesis of arterial thrombosis (coronary heart disease and stroke), it has been postulated that activated platelets may contribute to pulmonary inflammation and tissue destruction in CF, specifically (75). Certain specific pulmonary infections, such as *Burkholderia cepacia*, may also increase VTE risk in CF patients (76).

VTE in children with cystic fibrosis was recently studied at a single center and the incidence rate was found to be 53 VTE cases per 10,000 children with CF. Several inflammatory-specific risk factors for VTE were reported, including sinus disease, positive respiratory cultures, and elevated inflammatory markers such as erythrocyte sedimentation rate and CRP (7). A recent retrospective study of 116 adults with CF hospitalized for pulmonary exacerbations using a CVC demonstrated a 2.5% incidence of catheter-related VTE (77). Elevated CRP was associated with the thrombosis group but did not reach statistical significance (*p* = 0.51) and the time to VTE development was shorter for peripherally-inserted central catheter (PICC) lines compared to Port-a-Cath, though the only two recurrent events occurred in subjects with Port-a-Caths (77). A prospective study of 90 adult and pediatric CF patients, on the other hand, demonstrated a CVC-related VTE frequency of 6.6% (combination of symptomatic clots and those detected by screening ultrasound) (5). This study did not identify biomarkers (CRP, D-dimer, fibrinogen) for inflammatory/hypercoagulable screening at the time of catheter insertion, but did emphasize the contribution of VTE history and raises the issue of prospective Doppler ultrasound for consideration in identifying asymptomatic CVC-VTEs, though the role of anticoagulant therapy in the management of these patients remains controversial. The utility of pharmacologic prophylaxis for the prevention of CVC-associated thrombosis, particularly in high risk CF individuals, deserves further study (6).

## Sepsis and systemic infection

Bacterial sepsis is a classic example of inflammation-triggered coagulation induced by endothelial injury and tissue factor expression following an acute systemic inflammatory response, (78) leading to activation and consumption of coagulation factors and platelets, along with impaired fibrinolysis, disruption of endothelial barrier, and loss of physiologic antithrombotic factors such as thrombomodulin (79). Sepsis denotes progressively more severe host defense reactions to invading organisms with endothelial dysfunction and elevation of inflammatory markers triggering the activation of coagulation with concurrent down-regulation of anticoagulant systems and fibrinolysis (disseminated intravascular coagulation, DIC) which, in turn, contributes to increased inflammation. There is significant interplay between inflammation and coagulation in sepsis, highlighted by the procoagulant properties of the endothelium such as expression of tissue factor (TF) and VWF, activating interactions with platelets, release of procoagulant microparticles, and downregulation of TF pathway inhibitor (TFPI) and the protein C/S anticoagulant system, inhibiting fibrinolysis. The coagulation response serves to isolate the invasive organism, but some bacteria have adapted to this and can use the coagulation response to hide from immune attack (12, 46, 80).

As mentioned above regarding *B. cepacia* specifically imparting elevated VTE risk in CF patients, certain clinical variables and bacterial virulence factors are associated with VTE in *Staphylococcus aureus* bacteremia (Methicillin-resistance, CRP >20 mg/dL, and hemoglobin nadir </= 9 g/dL, though it may not always be clear whether anemia was an additional factor in the development of thrombus or is a marker of more severe disease) (11). These findings were comparable to those from another study of 70 children with osteomyelitis where in an elevated CRP was associated with increased VTE risk (81). Furthermore, a study of over 10,000 subjects in the Atherosclerosis Risk in Communities (ARIC) study, CRP above the 90th percentile was associated with a 76% increase in risk of VTE versus lower percentiles (24). A recent study of 39,831 patients who underwent colorectal surgery demonstrated a 2.4% incidence of VTE, associated with urinary tract infections, pneumonia, organ space surgical site infection, or deep surgical site infection (3).

## Systemic lupus erythematosus

In general, inflammatory rheumatologic diseases (lupus, Sjogren's syndrome, inflammatory myositis, and systemic sclerosis) are associated with high VTE rates—more than three times higher than in the general population (22). In particular, patients with systemic lupus erythematosus (SLE) are at significantly increased risk for premature atherosclerosis and thrombosis not only due to direct effects of chronic systemic inflammation but also to the additional risk imparted by antiphospholipid antibodies (21). This acquired, multi-organ, autoimmune disease itself is an independent risk factor for both arterial and venous thrombotic events, particularly in those with the antiphospholipid syndrome. Myriad factors influence atherosclerosis and cardiovascular disease in SLE patients, including those related to the disease itself (INF-alpha, TNF-alpha, MCP-1, cystatin C, kidney disease, overexpression of ICAM/VCAM/VEGF/VWF, NETs, elevations of interleukins 6/12/17/18, and other acute phase reactants) and to the medications used to treat it (corticosteroids, antimalarials, mycophenolate mofetil, HMG-CoA reductase inhibitors, and non-steroidal anti-inflammatories) (20). Individuals with SLE have been shown to have decreased DNase1 activity in their serum, which decreases NET degradation, and may contribute to thrombus formation (47). Activation of the innate immune system as evidenced by the elevation of TNF-alpha and the interleukins described above increases the risk of VTE even after adjustment for CRP (62). Polymorphisms in genes encoding factor VIII, Interleukin-1β, and interleukin-10 have been shown to modulate the risk of idiopathic VTE (82).

The lupus nephritis and autoimmune thrombocytopenia found in some of these patients also complicate the anticoagulant strategies and their standardization, but consistent APLA screening, renal evaluation/management, and use of hydroxycholorquine may decrease thrombotic risk or at least disease-related morbidity/mortality (19).

## Conclusion

Clinical observations and mounting laboratory evidence support a complex interplay between inflammation, innate immunity, and the coagulation system. As more is understood about these interactions, novel preventive and treatment modalities for thrombosis will likely become available.

## Author contributions

BB and SC contributed equally to the preliminary literature review, as well as the writing and revision of the manuscript.

### Conflict of interest statement

The authors declare that the research was conducted in the absence of any commercial or financial relationships that could be construed as a potential conflict of interest.
